# The differences in the number of fibroblasts and blood vessels after the topical and systemic administration of *Lactobacillus casei* Shirota probiotics for the treatment of traumatic ulcers in Wistar rats (*Rattus norvegicus*)

**DOI:** 10.14202/vetworld.2021.1279-1283

**Published:** 2021-05-23

**Authors:** Tuti Kusumaningsih, Anis Irmawati, Diah Savitri Ernawati, Chiquita Prahasanti, Mohammed Aljunaid, Sarah Amelia

**Affiliations:** 1 Department of Oral Biology, Faculty of Dental Medicine, Universitas Airlangga, Surabaya, Indonesia; 2 Department of Oral Medicine, Faculty of Dental Medicine, Universitas Airlangga, Surabaya, Indonesia; 3 Department of Periodontology, Faculty of Dental Medicine, Universitas Airlangga, Surabaya, Indonesia; 4 Postgraduate Program of Dental Medicine, Faculty of Dental Medicine, Universitas Airlangga, Surabaya, Indonesia; 5 Undergraduate Program of Dental Medicine, Faculty of Dental Medicine, Universitas Airlangga, Surabaya, Indonesia

**Keywords:** blood vessel, fibroblast, *Lactobacillus casei* Shirota, systemic administration, topical application, traumatic ulcers

## Abstract

**Background and Aim:**

The use of drugs as a therapy for traumatic ulcers may lead to drug resistance and other side effects. *Lactobacillus casei* Shirota can affect the number of fibroblasts and blood vessels in wound healing. The aim of this study was to investigate the difference in the number of fibroblast cells and blood vessels after the topical and systemic administration of *L. casei* Shirota probiotics in Wistar rats with traumatic ulcer.

**Materials and Methods:**

Overall, 36 healthy male Wistar rats aged 2-3 months old and weighing 175-250 g in body weight were used as a sample. Traumatic ulcer was made on the labial fornix incisive inferior. The subject rats were divided into groups: (1) A control group over 3 days, (2) a group that used distilled water over 7 days, (3) a group that underwent topical treatment over 3 days, (4) a group that used probiotics administered topically over 7 days, (5) a group that underwent systemic treatment over 3 days, and (6) a group that took oral probiotics for the traumatic ulcers over 7 days. The number of fibroblasts and blood vessels was observed through a hematoxylin-eosin examination.

**Results:**

Based on the results of the study, a significant difference was observed in the number of fibroblasts (p=0.00) and blood vessels (p=0.018) in the 3-day topical group that underwent a 3-day systemic administration of probiotics compared with the number of fibroblast cells in the 7-day topical group and 7-day systemic group (p=0.00).

**Conclusion:**

Overall, significant differences were observed in the number of fibroblasts and blood vessels in Wistar rats with traumatic ulcer after undergoing the topical and systemic administration of *L. casei* Shirota probiotics.

## Introduction

Oral traumatic ulcers occur when the oral epithelial tissue is damaged due to trauma. The prevalence rate of traumatic ulcers is 15-30% [1-3]. They can cause pain, discomfort, and malignancy when they are persistent [4]. The treatment of traumatic ulcers is usually performed through the administration of antiseptics, antibiotics, and steroids. However, antibiotics and steroids that are used for a long time can cause resistance and unpleasant side effects [5,6]. This led to the use of other alternative treatments, such as the use of probiotics.

Probiotics are defined as live microorganisms that, when administered in adequate amounts, can exhibit a positive effect on physical health [7,8]. One of the health benefits of probiotics, especially in the inflammatory process, is that they regulate the balance between T helper 1 as a producer of pro-inflammatory cytokines and T helper 2 as a producer of anti-inflammatory cytokines [9,10]. Probiotics are generally used in the form of lactic acid bacteria from the genus *Lactobacillus* and *Bifidobacterium*, such as *Bifidobacterium bifidum*, *Bifidobacterium breve*, *Lactobacillus acidophilus*, and *Lactobacillus casei*. *L. casei* Shirota is a strain of *Lactobacillus* spp., which is commonly used as a probiotic in the form of fermented milk [7,11].

The function of fibroblasts is to restore defects in the wound area by providing collagen in the new extracellular matrix and to produce a number of cytokines, chemokines, and growth factors in response to tissue damage [12]. Meanwhile, new blood vessels transport fluids, oxygen, nutrients, and immunocompetent cells during the healing process. The formation of blood vessels occurs with the involvement of endothelial cells and perivascular cells [13]. Probiotics, especially from the *Lactobacillus* spp. genus, can stimulate the migration and proliferation of fibroblasts and the formation of new blood vessels in the healing process of wounds [11].

However, only a few studies focused on the use of *L. casei* Shirota as a natural probiotic that is strain-specific. Thus, the aim of this study was to investigate the difference in the number of fibroblast cells and blood vessels after the administration of the probiotic *L. casei* Shirota topically and systemically during the onset of the healing of traumatic ulcers in Wistar rats (*Rattus norvegicus*).

## Materials and Methods

### Ethical approval

This study received the approval of ethical clearance from the Committee of Dental Medicine at Airlangga University, Indonesia (No. 336/HRECC.FODM/VII/2020).

### Study period and location

This study was conducted from July to October 2020 at the Research Center Faculty of Dental Medicine and Laboratorium of Biokimia Faculty of Medicine, University of Airlangga, Surabaya, Indonesia.

### Study design

This laboratory-based experimental study used 36 healthy male Wistar rats, aged 2-3 months, which presented with a body weight of approximately 175-250 g. Traumatic ulcer was created in the labial fornix incisive inferior by means of the heated tip of a round dental instrument. The subject rats were divided into six sample groups: 3-day control group, 7-day control group, 3-day topical treatment, 7-day topical treatment, 3-day systemic treatment, and 7-day systemic treatment. Each group consisted of six samples (n=6).

The control group, which included the rats with traumatic ulcers, was administered with sterile, distilled water up to 20 mL/20 g of body weight every day for 3 days and 7 days. The topical treatment group, which included the rats with traumatic ulcers, was administered with probiotics topically up to 10.9 × 10^7^ cells/kg body weight using the intraoral dropping method every day for 3- and 7-day periods and up to 10.9 × 10^7^ cells/kg body weight using oral gavage every day for 3- and 7-day periods [14-16]. The labial fornix incisive inferior tissue (mandibular labial mucosa) was taken on the 4^th^ and 8^th^ days after administering sterile, distilled water, and probiotics. The examination of fibroblasts and blood vessels was performed through hematoxylin-eosin staining before being counted under an inverted light microscope with 400×.

### Statistical analysis

Statistical analysis was conducted with analysis of variance test and *post* *hoc* test (Tukey HDS test), with a significance level (α =0.05) to compare the results of each treatment group.

## Results

The fibroblasts and blood vessels were counted using a light microscope with 400×. The results of the normality test, using the Kolmogorov–Smirnov test, showed that all groups were normally distributed (p>0.05), whereas the results of the homogeneity test, using Levene’s test, showed that all groups were homogeneous (p>0.05). The ANOVA test, along with the *post hoc* Tukey HSD test, was performed to compare the results of each treatment group (Tables-1 and 2).

**Table-1:** The result of the Tukey HSD test on fibroblast count between groups.

Group	Control day 3	Control day 7	Systemic day 3	Systemic day 7	Topical day 3	Topical day 7
Control day 3	---	0.544	0.039	0.00*	0.00*	0.00*
Control day 7	0.544	---	0.694	0.003*	0.00*	0.00*
Systemic day 3	0.039^*^	0.694	---	0.112	0.00*	0.00*
Systemic day 7	0.00^*^	0.003^*^	0.112	---	0.00*	0.00*
Topical day 3	0.00^*^	0.00^*^	0.00^*^	0.00^*^	---	0.544
Topical day 7	0.00^*^	0.00^*^	0.00^*^	0.00^*^	0.544	---

* Significant difference (p<0.05).

**Table-2:** The result of the Tukey HSD test on blood vessel count between groups.

Group	Control day 3	Control day 7	Systemic day 3	Systemic day 7	Topical day 3	Topical day 7
Control day 3	---	0.009*	0.00*	0.00*	0.00*	0.00*
Control day 7	0.009^*^	---	0.600	0.009*	0.00*	0.00*
Systemic day 3	0.00^*^	0.600	---	0.293	0.018*	0.001*
Systemic day 7	0.00^*^	0.009^*^	0.293	---	0.761	0.110
Topical day 3	0.00^*^	0.00^*^	0.018^*^	0.761	---	0.761
Topical day 7	0.00^*^	0.00^*^	0.001^*^	0.110	0.761	---

* Significant difference (p<0.05).

**Figure-1:**
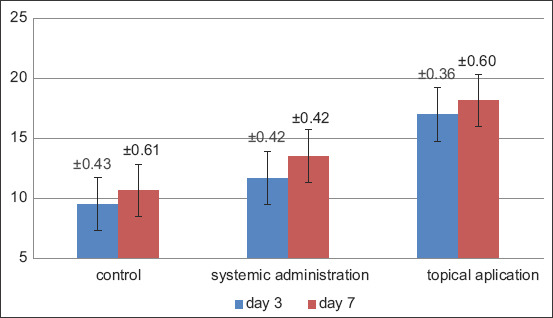
Graph of mean and standard deviation of fibroblast counts.

**Figure-2:**
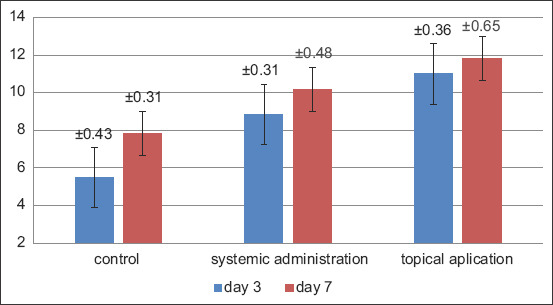
Graph of mean and standard deviation of blood vessel counts.

A significant improvement and difference were observed in the number of fibroblasts and blood vessels after the topical and systemic administration of probiotics for the treatment of traumatic ulcers in the subject animals. The present study showed that the topical administration of probiotics for 7 consecutive days produced the highest number of fibroblasts and blood vessels compared with the other groups, whereas the control group exhibited the lowest number of fibroblasts and blood vessels on the 3^rd^ day (Figures-1 and 2).

**Figure-3:**
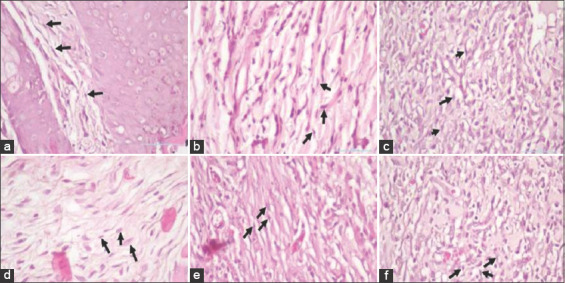
The fibroblast cell in (a) the control group on day 3, (b) control group on day 7, (c) topical group on day 3, (d) topical group on day 7, (e) systemic group on day 3, and (f) systemic group on day 7.

This study also demonstrated that topical administration is more effective in increasing the number of fibroblasts and blood vessels. This was proven in the topical administration group, which showed significantly different results compared with the control group and the 3- and 7-day systemic group, but no significant difference was observed in the topical administration for 3 or 7 days (Figure-3).

## Discussion

The wound healing process is a dynamic process that consists of several phases: Inflammation, proliferation, maturation, and remodeling. Fibroblasts and blood vessels play an important role in the proliferation phase, primarily to correct defects in the wound area by providing collagen to the new extracellular matrix, which is supported by the presence of fluid, oxygen, nutrients, and immunocompetent cells that are transported by new blood vessels [17].

In the present study, a significant increase was observed in the number of fibroblasts and blood vessels after the administration of probiotics both topically and systemically for 3- and 7-day periods when compared with the control group. This finding indicated that the administration of probiotics to the wound area can accelerate the healing process, as probiotics can increase the number of blood vessels and fibroblasts. Several studies reported that the application of probiotics, especially from *Lactobacillus* spp. genus, can stimulate the migration and proliferation of fibroblasts as well as induce the production of interleukin (IL)-8, which can stimulate the migration of endothelial cells for the process of forming new blood cells [11,18].

Probiotics are better known as a product that is consumed orally, but the topical administration of probiotics is increasingly being developed as a treatment. Probiotics can act as a modulator to restore the microbial balance through topical administration when dysbiosis occurs on the skin. Probiotics can be combined with keratin cells, which can activate a pathway for probiotics to provide a beneficial mechanism of action for the host, that is, through the activation of a toll-like receptor (TLR) [10]. *L. casei* Shirota is a Gram-positive bacterium whose cell wall contains an active molecular component of lipoteichoic acid (LTA), which is a pathogen-associated molecular pattern *L. casei* Shirota acts as an inducer, whereas LTA activates the oral mucosal epithelial cells through the TLR-2. This TLR activation induces the production of chemokines and cytokines, such as IL-8, which will stimulate the reepithelialization process, angiogenesis, and the formation of the extracellular matrix [11,19,20].

In comparison, the systemic administration of probiotics interacts with and attaches to the gastrointestinal mucosa and gut-associated lymphoid tissue, which is an important component of the immune cells [21]. In the inflammatory process, following the interaction between the probiotic bacteria and the gastrointestinal mucosa, the TLR pathway is activated. The activation of the TLR pathway can inhibit the production of pro-inflammatory cytokines, increase the gastrointestinal mucosal barrier, and modulate the immune system [11,22].

In addition to this mechanism, the probiotic and commensal bacteria in the digestive system exhibit a microbiome-gut-brain axis mechanism in which the neurotransmitter, in the form of oxytocin, is mediated through the vagus nerve to the central nervous system or through the blood vessels, to influence the body’s immune system. The administration of probiotics can improve the healing process of wounds by increasing the regulation of oxytocin in the microbiome-gut-brain axis, which is transmitted through the vagus nerve’s pathway. The improvement of systemic oxytocin regulation may upregulate keratinocyte and fibroblast activity so that the formation of the extracellular matrix can occur [11,22,23].

The results of this study showed that the topical administration of probiotics was more effective in increasing the number of fibroblast cells and blood vessels in the healing process of the wound due to the probiotic adhesion that took place directly on the wound area.

The topical application of probiotics may be more effective for the healing process of wounds. This is in accordance with research conducted by Cervin [24] whom reported that the topical administration of probiotics is a more effective therapy compared with the systemic administration of probiotics because, in the topical administration, the combination of mechanisms between the intervention of probiotic bacteria and the stimulation of the immune system is beneficial for the healing process. Moreover, probiotics that are administered topically will also naturally go to the digestive system, which allow the interactions between probiotic bacteria and lymphoid tissue in the digestive system. Through the modulation of the immune system, this will exhibit additional effects on the wound’s healing process [24].

## Conclusion

Overall, significant differences were observed in the number of fibroblasts and blood vessels after the topical and systemic administration of *L. casei* Shirota in the healing process of traumatic ulcers in Wistar rats. Furthermore, the topical administration of *L. casei* Shirota is more effective than systemic administration. Therefore, the topical administration of *L. casei* Shirota is recommended for the acceleration of the healing process of traumatic ulcers.

## Authors’ Contributions

TK, AI, DSE, CP., MA, and SA designed the study, wrote the manuscript, and participated in conducting the experiment. TK and SA performed the *in vivo* experiment and collected the samples and performed the histological investigations. AI and MA processed and analyzed the data. All authors read and approved the final manuscript.
